# Associations between dietary fiber intake and mortality from all causes, cardiovascular disease and cancer: a prospective study

**DOI:** 10.1186/s12967-022-03558-6

**Published:** 2022-08-02

**Authors:** Xin Xu, Junmiao Zhang, Yanhui Zhang, Honggang Qi, Ping Wang

**Affiliations:** 1 Department of Urology, First Affiliated Hospital, School of Medicine, Zhejiang University, 79 Qingchun Road, Hangzhou, 310003 Zhejiang China; 2 Department of Urology, The Second Affiliated Hospital of Medical College of Qingdao University, Qingdao, 266042 Shandong China

**Keywords:** Fiber, Cardiovascular disease, Cancer, Cohort, PLCO

## Abstract

**Objective:**

Several studies suggest that dietary fiber intake may reduce mortality risk, but this might depend on the fiber types and the evidence regarding the role of soluble fiber or insoluble fiber on death risk remain limited and inconsistent. Therefore, this study aimed to comprehensively evaluate multiple types of dietary fiber intake on mortality from all causes, cardiovascular disease and cancer in the large-scale Prostate, Lung, Colorectal, and Ovarian Cancer (PLCO) Screening Trial.

**Methods:**

A multivariate Cox proportional hazards model was used to estimate hazard ratios (HRs) and 95% confidence intervals (CIs).

**Results:**

This study finally included 86,642 participants with 17,536 all-cause deaths, 4842 cardiovascular deaths and 5760 cancer deaths identified after a total of 1,444,068 follow-up years. After adjusting for potential confounders, dietary total fiber intake was statistically significantly inversely associated with all-cause death (Q5 vs Q1: HR 0.71, 95% CI 0.66–0.75; *P* for trend < 0.001), cardiovascular death (Q5 vs Q1: HR 0.73, 95% CI 0.65–0.83; *P* for trend < 0.001) and cancer mortality (Q5 vs Q1: HR 0.77, 95% CI 0.69–0.86; *P* for trend < 0.001). Similar results were observed for both insoluble and soluble fiber intake. Restricted cubic spline model analysis suggested that there was a nonlinear association of dietary fiber intake with mortality risk (all *P* for nonlinearity < 0.05).

**Conclusions:**

In this large nationally representative sample of US adult population, intakes of total fiber, soluble fiber, and insoluble fiber were associated with lower risks of all-cause, cardiovascular and cancer mortality.

**Supplementary Information:**

The online version contains supplementary material available at 10.1186/s12967-022-03558-6.

## Introduction

Non-communicable diseases (NCDs) continue to be important public health problems in the world as the leading cause of death globally, responsible for approximately 70% of mortality worldwide [1]. The majority of these deaths were due to cardiovascular disease, cancer, chronic respiratory diseases and diabetes. Unhealthy diet is an important modifiable risk factor for NCDs [2]. A recent study from the Global Burden of Diseases (GBD) consortium reported that 11 million deaths were attributable to dietary risk factors [3]. High intake of sodium, and low intake of whole grains, and fruits were the leading dietary risk factors for death globally [3].

Whole grains and fresh fruits are the major sources of dietary fiber [4]. Inadequate consumption of dietary fiber has been associated with a variety of health outcomes, including cancer (e.g., colorectal cancer [5], breast cancer [6], endometrial cancer [7], and renal cell carcinoma [8]), diabetes [9], and CVD [10]. Emerging data also have indicated a potential inverse associations of total dietary fiber intake with all-cause or cause-specific mortality [11–13]. However, controversy still exists [14, 15] and there are some differences for men and women [12]. In addition, there are two main types of fiber: insoluble fiber and soluble fiber. Soluble fiber is found in oat bran, barley, beans, lentils, peas, and some fruits and vegetables. Insoluble fiber is rich in foods such as wheat bran, whole grains, nuts, and seeds. Previous studies suggest that dietary fiber may be differentially associated with health outcomes depending on their solubility [16, 17]. For instance, soluble fiber is prompted as an important part of stabilizing blood sugar and improving insulin responses [18]. Intake of soluble fiber supplementation is effective in improving glycemic control in type 2 diabetes [19]. In contrast, insoluble fiber is characterized by a fecal-bulking ability, which may reduce the risk of colon cancer [20]. Currently, the body of evidence regarding soluble fiber or insoluble fiber remain limited and inconsistent.

In this context, our objectives were to investigate the associations between intake of dietary fiber of different types (i.e., total fiber, soluble fiber and insoluble fiber) and mortality from all causes, cardiovascular disease and cancer in a large prospective cohort of US adults.

## Methods

### Study population

Study participants were identified from the Prostate, Lung, Colorectal, and Ovarian Cancer (PLCO) Screening Trial. The design and methods of the PLCO trial have been previously described [21]. Briefly, the PLCO study is a randomized, controlled trial to determine whether certain screening tests reduce death from prostate, lung, colorectal, and ovarian cancer. PLCO consisted of 154,952 individuals aged 55 to 74 years and enrolled between November 1993 and July 2001. The participants were recruited via 10 centers in the United States. These PLCO Screening Centers recruited possible participants and evaluated their eligibility to participate in PLCO. Nine of the ten centers began enrollment in November 1993. The tenth center began enrollment in January 1998. All participants provided written informed consent, and the study was approved by the Institutional Review Boards at the National Cancer Institute and each of the participating centers.

### Data collection and dietary assessment

All participants were asked to complete a baseline questionnaire (BQ) containing baseline information such as demographics, medical history and other selected life style factors. The Dietary History Questionnaire (DHQ) was administered to participants to collect dietary data. 77% of all participants in both arms of the trial completed the DHQ. The form was introduced 5 years into the trial (December 1998). Raw questionnaire responses were processed into analysis-ready variables in terms of gram intake, pyramid servings, food frequencies per day, to name just a few. DHQ included the prespecified portion size and consumption frequency of 124 food items and supplement use over the previous year [22]. The USDA 1994 to 1996 Continuing Survey of Food Intakes by Individuals [23] were used to calibrate DHQ data and calculate the daily fiber intake. Main sources of dietary fiber were cereal/grain, vegetables, fruit, and legumes [24].

### Participant selection

Participants were excluded from this study if they did not complete a BQ (n = 4918); had reported a previous cancer (n = 10,199), heart disease (n = 12,616), stroke (n = 2410), or diabetes (n = 8076) at baseline; did not have follow-up time (n = 13); failed to complete DHQ or the DHQ was not valid (n = 30,023). Thus, this study included 86,642 participants. The main characteristics of participants included and excluded are shown in Additional file 1: Table S1.

### Outcome assessment

Participants were followed from the date of DHQ completion to the time of death or through 2015. Vital status was obtained by the administration of the Annual Study Update questionnaire, reports from relatives, friends, or physicians, and National Death Index. Study centers attempted to obtain a death certificate for each death. The cause of deaths was classified according to the International Classification of Diseases, 9th Revision (ICD-9). The primary outcomes of interest were all-cause mortality (death from any cause), and mortality from CVD or cancer.

### Statistical analysis

Dietary fiber intake was categorized into five equal groups. Cox proportional hazards models were used to estimate the hazard ratios (HRs) and corresponding 95% confidence intervals (CIs) for the mortality risk associated with fiber intake. Model included adjustment for age (continuous), sex (male vs. female), race (non-Hispanic White vs. Other), body mass index (BMI, < 25.0 kg/m^2^ vs. ≥ 25.0 kg/m^2^), education (≤ high school vs. ≥ some college), smoking status (never vs. former ≤ 15 years since quit vs. former > 15 years since quit vs. former year since quit unknown vs. current smoker ≤ 1 pack per day vs. current smoker > 1 pack per day vs. current smoker intensity unknown), marital status (married vs. not married), alcohol drinking status (never vs. former vs. current), and total energy intake (continuous). Tests for trend were assessed by assigning each individual in a particular quintile of fiber intake the median value for that quintile.

Stratified analyses were performed based on age, sex, race, smoking status, drinking habits, education level and BMI. Sensitivity analyses were conducted by excluding events that occurred within 2 years or within 5 years of follow-up. Interactions were examined by using likelihood-ratio tests. The proportional hazards (PH) assumption was checked using the Schoenfeld residual test [25]. Restricted cubic spline models [26] with three fitted knots (i.e., 10th, 50th, and 90th percentiles) were used to investigate the dose–response relationship between dietary fiber intake (as a continuous variable) and each outcome after full adjustment. A 2-tailed *P* value < 0.05 was considered significant, and analyses were conducted by using STATA version 15 (Stata Corp, College Station, TX, USA).

## Results

### Cohort characteristics

During a total of 1,444,068 follow-up years, 17,536 all-cause deaths, 4842 cardiovascular deaths and 5760 cancer deaths were identified. The median (IQR) follow-up duration was 17.1 (15.3–19.1) years. The average age of participants at baseline was 62.1 (SD 5.2) years. The median (IQR) intakes of dietary fiber were 16.5 (12.1–22.1) g/day. In comparison with participants in the lowest category of dietary fiber intake, participants in the highest category were more often female, married, and tended to have a higher level of education, and less often be current smokers (Table 1).

**Table 1:** Main characteristics of participants included in this study by dietary fiber intake

Variables	Q1 (*n* = 17,360)	Q2 (*n* = 17,330)	Q3 (*n* = 17,322)	Q4 (*n* = 17,304)	Q5 (*n* = 17,326)	*p*
Age (years), mean (SD)	62.1 (5.2)	62.2 (5.2)	62.1 (5.2)	62.1 (5.2)	62.1 (5.2)	0.35
Sex (*n*, %)						
Female	6579 (37.9%)	7041 (40.6%)	7661 (44.2%)	8469 (48.9%)	9931 (57.3%)	** < 0.001**
Male	10,781 (62.1%)	10,289 (59.4%)	9661 (55.8%)	8835 (51.1%)	7395 (42.7%)	
Arm (*n*, %)						
Screen	8747 (50.4%)	8808 (50.8%)	8686 (50.1%)	8998 (52.0%)	8968 (51.8%)	** < 0.001**
Control	8613 (49.6%)	8522 (49.2%)	8636 (49.9%)	8306 (48.0%)	8358 (48.2%)	
Smoking (*n*, %)						
Never	7998 (46.1%)	8493 (49.0%)	8664 (50.0%)	8816 (51.0%)	8698 (50.2%)	** < 0.001**
Current	2451 (14.1%)	1726 (10.0%)	1465 (8.5%)	1249 (7.2%)	1168 (6.7%)	
Former	6909 (39.8%)	7107 (41.0%)	7192 (41.5%)	7231 (41.8%)	7457 (43.0%)	
Education (*n*, %)						
≤ High school	8187 (47.2%)	7393 (42.7%)	7026 (40.6%)	6765 (39.1%)	6492 (37.5%)	** < 0.001**
≥ Some college	9126 (52.6%)	9911 (57.2%)	10,272 (59.3%)	10,494 (60.6%)	10,801 (62.3%)	
BMI (*n*, %)						
< 25.0 kg/m^2^	6169 (35.5%)	6264 (36.1%)	6217 (35.9%)	6190 (35.8%)	6155 (35.5%)	0.75
≥ 25.0 kg/m^2^	10,942 (63.0%)	10,842 (62.6%)	10,879 (62.8%)	10,903 (63.0%)	10,944 (63.2%)	
Race (*n*, %)						
White, Non-Hispanic	15,530 (89.5%)	15,932 (91.9%)	16,071 (92.8%)	16,045 (92.7%)	15,703 (90.6%)	** < 0.001**
Other	1830 (10.5%)	1398 (8.1%)	1251 (7.2%)	1259 (7.3%)	1623 (9.4%)	
Marital status (*n*, %)						
Married	12,815 (73.8%)	13,622 (78.6%)	13,830 (79.8%)	13,845 (80.0%)	13,726 (79.2%)	** < 0.001**
Not married	4505 (26.0%)	3685 (21.3%)	3466 (20.0%)	3412 (19.7%)	3571 (20.6%)	

*y* year, *SD* Standard deviation, *BMI* body mass index

### Dietary fiber intake and all-cause mortality

HRs for all-cause mortality across total dietary fiber quintiles are presented in Table 2. After adjusting for confounders, dietary total fiber intake was statistically significantly inversely associated with all-cause mortality (Q5 vs Q1: HR 0.71, 95% CI 0.66–0.75; *P* for trend < 0.001). When fiber was analyzed as a continuous variable, the results were similar. Corresponding adjusted HR was 0.89 (95% CI 0.87–0.91) per 1 SD increment of dietary fiber intake. Similar patterns of associations were observed for insoluble fiber (Q5 vs Q1: HR 0.71, 95% CI 0.67–0.75; *P* for trend < 0.001) and soluble fiber (Q5 vs Q1: HR 0.76, 95% CI 0.71–0.81; *P* for trend < 0.001), separately.

**Table 2:** Association between dietary fiber intake and all-cause mortality

Variables	Median (g/day)	Cohort (*n*)	Cases (*n*)	Crude HR (95% CI), *p*-value	Adjusted HR* (95% CI), *p*-value
Total					
Q1 (< 11.12)	8.83	17,360	3852	Reference	Reference
Q2 (≥ 11.12 to < 14.71)	12.95	17,330	3515	0.88 (0.84–0.93), **p < 0.001**	0.88 (0.84–0.92), **p < 0.001**
Q3 (≥ 14.71 to < 18.41)	16.48	17,322	3380	0.83 (0.79–0.87), **p < 0.001**	0.82 (0.78–0.86), **p < 0.001**
Q4 (≥ 18.41 to < 23.75)	20.74	17,304	3348	0.82 (0.78–0.85), **p < 0.001**	0.77 (0.73–0.82), **p < 0.001**
Q5 (≥ 23.75)	28.75	17,326	3441	0.83 (0.79–0.87), **p < 0.001**	0.71 (0.66–0.75), **p < 0.001**
				**p for trend < 0.001**	**p for trend < 0.001**
Insoluble					
Q1 (< 7.21)	5.7	17,366	3871	Reference	Reference
Q2 (≥ 7.21 to < 9.63)	8.44	17,342	3554	0.89 (0.85–0.93), **p < 0.001**	0.89 (0.85–0.93), **p < 0.001**
Q3 (≥ 9.63 to < 12.11)	10.8	17,312	3361	0.82 (0.78–0.86), **p < 0.001**	0.82 (0.78–0.86), **p < 0.001**
Q4 (≥ 12.11 to < 15.73)	13.69	17,332	3318	0.80 (0.77–0.84), **p < 0.001**	0.77 (0.73–0.81), **p < 0.001**
Q5 (≥ 15.73)	19.09	17,290	3432	0.82 (0.78–0.86), **p < 0.001**	0.71 (0.67–0.75), **p < 0.001**
				**p for trend < 0.001**	**p for trend < 0.001**
Soluble fiber					
Q1 (< 3.72)	2.97	17,395	3817	Reference	Reference
Q2 (≥ 3.72 to < 4.91)	4.32	17,304	3477	0.89 (0.85–0.93), **p < 0.001**	0.89 (0.85–0.93), **p < 0.001**
Q3 (≥ 4.91 to < 6.15)	5.49	17,351	3404	0.85 (0.81–0.89), **p < 0.001**	0.84 (0.80–0.88), **p < 0.001**
Q4 (≥ 6.15 to < 7.92)	6.91	17,266	3290	0.81 (0.77–0.85), **p < 0.001**	0.77 (0.73–0.81), **p < 0.001**
Q5 (≥ 7.92)	9.62	17,326	3548	0.87 (0.83–0.91), **p < 0.001**	0.76 (0.71–0.81), **p < 0.001**
				**p for trend < 0.001**	**p for trend < 0.001**

^*^Adjusted for age (continuous), sex (male vs. female), race (non-Hispanic White vs. Other), body mass index (BMI, < 25.0 kg/m^2^ vs. ≥ 25.0 kg/m^2^), education (≤ high school vs. ≥ some college), smoking status (never vs. former ≤ 15 years since quit vs. former > 15 years since quit vs. former year since quit unknown vs. current smoker ≤ 1 pack per day vs. current smoker > 1 pack per day vs. current smoker intensity unknown), marital status (married vs. not married), alcohol drinking status (never vs. former vs. current), and total energy intake (continuous)

### Dietary fiber intake and cause-specific mortality

As can be seen from Table 3, based on the fully adjusted model, higher intake of dietary total fiber was statistically significantly associated with a lower risk of cardiovascular mortality (Q5 vs Q1: HR 0.73, 95% CI 0.65–0.83; *P* for trend < 0.001)(Table 3). Greater consumption of insoluble fiber (Q5 vs Q1: HR 0.72, 95% CI 0.65–0.81; *P* for trend < 0.001) and soluble fiber (Q5 vs Q1: HR 0.78, 95% CI 0.69–0.88; *P* for trend < 0.001) were also significantly associated with a lower risk of cardiovascular death.

**Table 3:** Association between dietary fiber intake and CVD mortality

Variables	Median (g/day)	Cohort (n)	Cases (n)	Crude HR (95% CI), *p*-value	Adjusted HR* (95% CI), *p*-value
Total					
Q1 (< 11.12)	8.83	17,360	1044	Reference	Reference
Q2 (≥ 11.12 to < 14.71)	12.95	17,330	987	0.91 (0.84–1.00), **p = 0.044**	0.91 (0.84–1.00), **p = 0.046**
Q3 (≥ 14.71 to < 18.41)	16.48	17,322	931	0.84 (0.77–0.92), **p < 0.001**	0.83 (0.76–0.92), **p < 0.001**
Q4 (≥ 18.41 to < 23.75)	20.74	17,304	913	0.82 (0.75–0.89), **p < 0.001**	0.78 (0.70–0.86), **p < 0.001**
Q5 (≥ 23.75)	28.75	17,326	967	0.85 (0.78–0.93), **p < 0.001**	0.73 (0.65–0.83), **p < 0.001**
				**p for trend < 0.001**	**p for trend < 0.001**
Insoluble					
Q1 (< 7.21)	5.7	17,366	1055	Reference	Reference
Q2 (≥ 7.21 to < 9.63)	8.44	17,342	1003	0.92 (0.84–1.00), p = 0.056	0.92 (0.84–1.00), p = 0.063
Q3 (≥ 9.63 to < 12.11)	10.8	17,312	920	0.82 (0.75–0.90), **p < 0.001**	0.82 (0.75–0.90), **p < 0.001**
Q4 (≥ 12.11 to < 15.73)	13.69	17,332	899	0.79 (0.73–0.87), **p < 0.001**	0.76 (0.69–0.84), **p < 0.001**
Q5 (≥ 15.73)	19.09	17,290	965	0.84 (0.77–0.92), **p < 0.001**	0.72 (0.65–0.81), **p < 0.001**
				**p for trend < 0.001**	**p for trend < 0.001**
Soluble fiber					
Q1 (< 3.72)	2.97	17,395	1059	Reference	Reference
Q2 (≥ 3.72 to < 4.91)	4.32	17,304	930	0.85 (0.78–0.93), **p < 0.001**	0.87 (0.79–0.95), **p = 0.002**
Q3 (≥ 4.91 to < 6.15)	5.49	17,351	945	0.85 (0.78–0.93), **p < 0.001**	0.85 (0.77–0.93), **p = 0.001**
Q4 (≥ 6.15 to < 7.92)	6.91	17,266	914	0.81 (0.74–0.88), **p < 0.001**	0.78 (0.71–0.86), **p < 0.001**
Q5 (≥ 7.92)	9.62	17,326	994	0.88 (0.80–0.96), **p = 0.003**	0.78 (0.69–0.88), **p < 0.001**
				**p for trend = 0.011**	**p for trend < 0.001**

^*^Adjusted for age (continuous), sex (male vs. female), race (non-Hispanic White vs. Other), body mass index (BMI, < 25.0 kg/m^2^ vs. ≥ 25.0 kg/m^2^), education (≤ high school vs. ≥ some college), smoking status (never vs. former ≤ 15 years since quit vs. former > 15 years since quit vs. former year since quit unknown vs. current smoker ≤ 1 pack per day vs. current smoker > 1 pack per day vs. current smoker intensity unknown), marital status (married vs. not married), alcohol drinking status (never vs. former vs. current), and total energy intake (continuous)

In Table 4, a lower risk of cancer mortality was observed for higher intake of dietary total fiber (Q5 vs Q1: HR 0.77, 95% CI 0.69–0.86; *P* for trend < 0.001). Similar patterns were observed for both insoluble fiber (Q5 vs Q1: HR 0.79, 95% CI 0.71–0.87; *P* for trend < 0.001) and soluble fiber (Q5 vs Q1: HR 0.79, 95% CI 0.71–0.88; *P* for trend < 0.001).

**Table 4:** Association between dietary fiber intake and cancer mortality

Variables	Median (g/day)	Cohort (n)	Cases (n)	Crude HR (95% CI), *p*-value	Adjusted HR* (95% CI), *p*-value
Total					
Q1 (< 11.12)	8.83	17,360	1247	Reference	Reference
Q2 (≥ 11.12 to < 14.71)	12.95	17,330	1150	0.90 (0.83–0.97), **p = 0.009**	0.91 (0.84–0.99), **p = 0.024**
Q3 (≥ 14.71 to < 18.41)	16.48	17,322	1114	0.85 (0.79–0.93), **p < 0.001**	0.86 (0.79–0.94), **p = 0.001**
Q4 (≥ 18.41 to < 23.75)	20.74	17,304	1109	0.85 (0.78–0.92), **p < 0.001**	0.83 (0.76–0.91), **p < 0.001**
Q5 (≥ 23.75)	28.75	17,326	1140	0.86 (0.79–0.93), **p < 0.001**	0.77 (0.69–0.86), **p < 0.001**
				**p for trend < 0.001**	**p for trend < 0.001**
Insoluble					
Q1 (< 7.21)	5.7	17,366	1254	Reference	Reference
Q2 (≥ 7.21 to < 9.63)	8.44	17,342	1166	0.91 (0.84–0.98), **p = 0.015**	0.93 (0.86–1.01), p = 0.075
Q3 (≥ 9.63 to < 12.11)	10.8	17,312	1099	0.84 (0.77–0.91), **p < 0.001**	0.85 (0.78–0.93), **p < 0.001**
Q4 (≥ 12.11 to < 15.73)	13.69	17,332	1095	0.83 (0.76–0.90), **p < 0.001**	0.83 (0.75–0.91), **p < 0.001**
Q5 (≥ 15.73)	19.09	17,290	1146	0.86 (0.79–0.93), **p < 0.001**	0.79 (0.71–0.87), **p < 0.001**
				**p for trend < 0.001**	**p for trend < 0.001**
Soluble fiber					
Q1 (< 3.72)	2.97	17,395	1224	Reference	Reference
Q2 (≥ 3.72 to < 4.91)	4.32	17,304	1161	0.93 (0.86–1.01), p = 0.073	0.93 (0.86–1.01), p = 0.099
Q3 (≥ 4.91 to < 6.15)	5.49	17,351	1109	0.87 (0.80–0.95), **p = 0.001**	0.87 (0.79–0.95), **p = 0.001**
Q4 (≥ 6.15 to < 7.92)	6.91	17,266	1111	0.86 (0.80–0.94), **p < 0.001**	0.83 (0.76–0.91), **p < 0.001**
Q5 (≥ 7.92)	9.62	17,326	1155	0.90 (0.83–0.97), **p = 0.009**	0.79 (0.71–0.88), **p < 0.001**
				**p for trend = 0.008**	**p for trend < 0.001**

^*^Adjusted for age (continuous), sex (male vs. female), race (non-Hispanic White vs. Other), body mass index (BMI, < 25.0 kg/m^2^ vs. ≥ 25.0 kg/m^2^), education (≤ high school vs. ≥ some college), smoking status (never vs. former ≤ 15 years since quit vs. former > 15 years since quit vs. former year since quit unknown vs. current smoker ≤ 1 pack per day vs. current smoker > 1 pack per day vs. current smoker intensity unknown), marital status (married vs. not married), alcohol drinking status (never vs. former vs. current), and total energy intake (continuous)

### Additional analyses

**Fig. 1:**
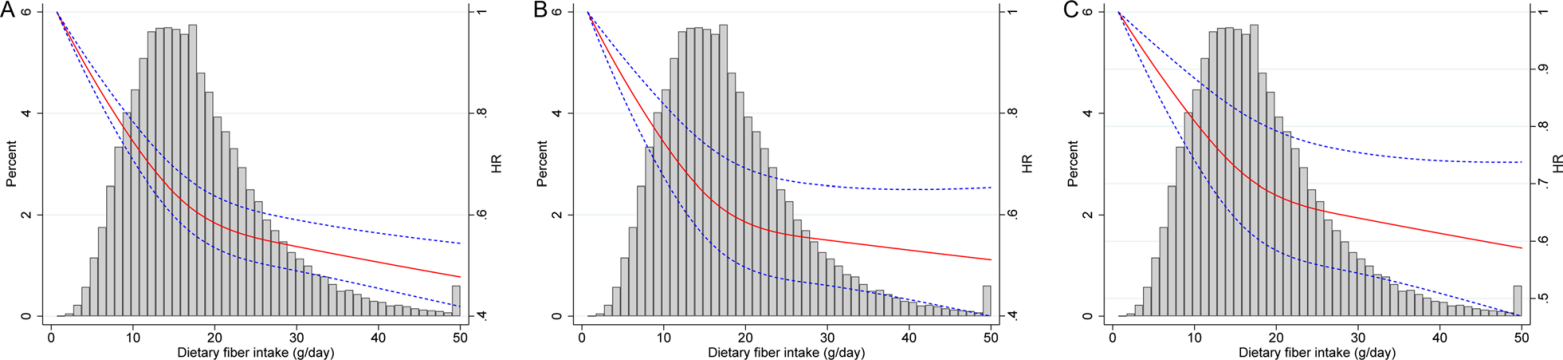
Dose–response using restricted cubic spline model for the association between total fiber intake and mortality from all causes (**A**), cardiovascular disease (**B**) and cancer (**C**). Solid line represents point estimates and dashed lines represent 95% confidence intervals. Multivariable risk estimate was calculated by restricted cubic spline regression (using 3 knots at 10th, 50th, and 90th percentiles) adjusting for age, sex, race, body mass index, education, smoking status, marital status, alcohol drinking status, and total energy intake. The histograms show the percentage of participants (left y axis) consuming each level of fiber

**Fig. 2:**
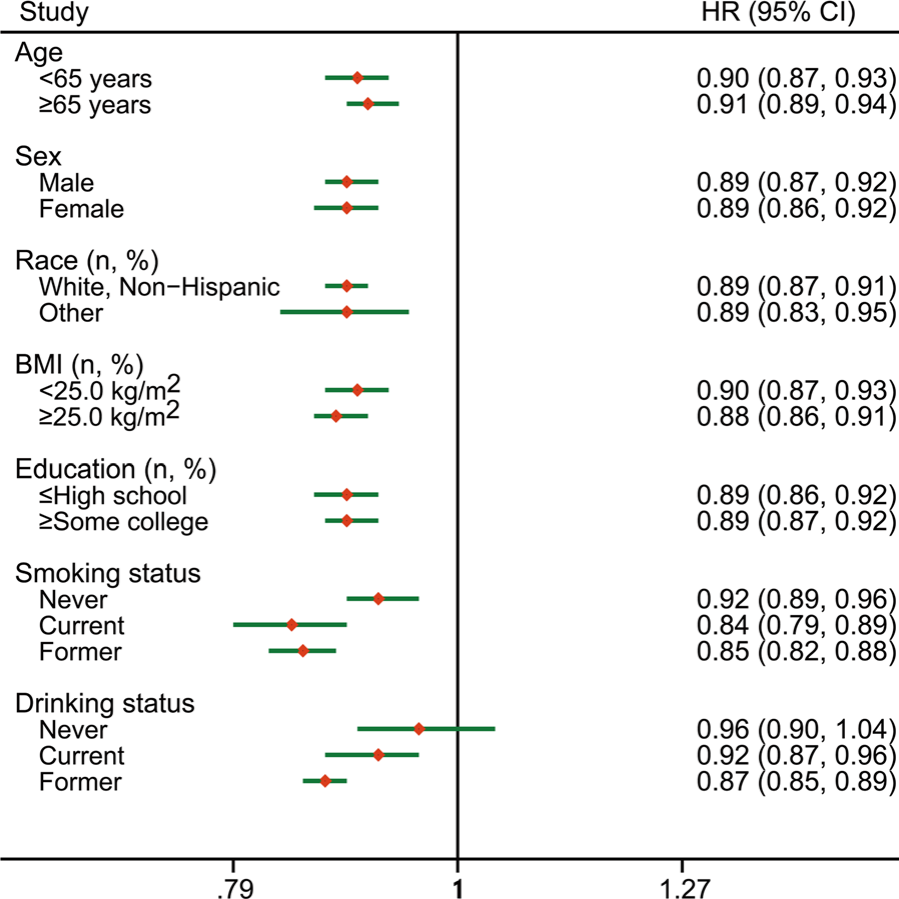
Subgroup analyses by potential confounders including age (< 65 years vs. ≥ 65 years), sex (male vs. female), race (White, Non-Hispanic vs. Other), body mass index at the time of enrollment (< 25 kg/m^2^ vs. ≥ 25 kg/m^2^), education (≤ high school vs. ≥ some college), smoking status (never vs. former vs. current), and drinking status (never vs. former vs. current). The HRs (95% CIs) of per SD increment in the total fiber intake were calculated and showed. *HRs* hazard ratios; *CIs* confidence intervals; *SD* standard deviation

Restricted cubic spline model analysis suggested that there was a nonlinear association of dietary fiber intake with deaths from all causes, cardiovascular disease and cancer (Fig. 1, all *P* for nonlinearity < 0.05). The results of subgroup analyses are presented in Fig. 2. Dietary total fiber intake was consistently associated with reduced risk of all-cause mortality in all subgroups, except for those who were never alcohol drinkers. In a sensitivity analysis, results remained qualitatively similar after excluding events ascertained within 2 or 5 years (data not shown).

## Discussion

In this large prospective cohort of US adults, a higher intake of dietary fiber was associated with reduced risk of mortality from all causes, cardiovascular disease and cancer. This was equally true for total dietary fiber, soluble fiber, and insoluble fiber. The results were qualitatively similar for men and women. Restricted cubic spline model analysis suggested that there was a nonlinear association between dietary fiber intake and mortality.

Our findings were consistent with previous meta-analyses of prospective studies that suggested that higher intakes of dietary fibers were associated with decreased risk of mortality from all causes, cardiovascular disease and cancer [16, 27–29]. However, the body of evidence regarding soluble fiber and insoluble fiber remain limited and inconsistent so far [11]. For instance, although 25 eligible studies were included in Liu et al.’s meta-analysis, only three studies were available for subgroup analysis according to fiber types (i.e., soluble and insoluble fiber), and these studies had conflicting results with limited sample size [29]. The findings of our study enriched evidence on this topic and supported a potential benefit of both soluble and insoluble fiber in the prevention of death. Similarly, Arayici et al. [30] also found that both soluble and insoluble fiber consumption were protective against colorectal cancer, with a clinically significant reduction in colorectal cancer risk based on a large meta-analysis.

Several potential mechanisms could explain the beneficial effects of dietary fiber intake on health outcome, including stabilizing blood sugar, improving insulin responses, lowering levels of inflammatory biomarkers (e.g., C-reactive protein and interleukin-6), and reducing total and low density lipoprotein cholesterol [18, 31–34]. In addition, the potential protective role of dietary fiber on prevention of chronic diseases could be mediated by the production of short-chain fatty acids (SCFAs), as a result of fermentation of undigestible fiber by gut microbiota [35, 36]. SCFAs may play a role on maintaining the metabolic health of the human host, as key regulators of anti-inflammatory effects [37–39].

The average dietary fiber intake (16.5 g/d) in this cohort was still far below the recommended level worldwide as 30 g/d of total fiber intake. Likewise, García-Meseguer et al. [40] investigated the fiber patterns in youngsters from three different counties (US, Spain, and Tunisia) and found that the mean fiber intake was only 17.8 g/day. Similarly, Casagrande et al. [41] found that fiber intake significantly decreased over time and remained below the recommendation level among type 2 diabetes patients based on the data of NHANES 1988–2012. In Asian population, Nakaji et al. [42] reported a decline in total dietary fiber intake in Japan using data compiled in the Japanese National Nutrition Survey. Therefore, improving fiber intake is a promising target for public health, with appropriate actions needed to increase the intake of dietary fiber through a large variety of sources in the population. We hope our findings, as along with others, would help clinicians, policymakers, and others make informed decisions about the provision of health care interventions in order to raise the population’s awareness of the health benefit of fiber intake and promote the consumption of foods rich in fiber in public health practice [43].

The major strengths of this study included a large sample size, a prospective design, long-term follow-up, detailed information on diet and potential risk factors of death, and available data on both soluble and insoluble fiber intake. However, as with any study, there were some limitations in this study. First, dietary fiber intake might be a surrogate for a healthy lifestyle. Although we have adjusted for various lifestyle factors in the multivariable model, residual confounding cannot be fully ruled out. Second, participants analyzed in this study were mainly non-Hispanic Whites, which may limit the generalizability of our findings to other population. Third, the sources of dietary fiber (e.g., fruit, vegetables, legumes and cereals) were not available and thus we were unable to perform stratified analyses according to fiber sources. Fourth, previous studies have suggested that dietary fiber intake reduced the risk of pancreatic cancer, colon cancer, and rectal cancer [44–46]. Unfortunately, we were unable to perform analyses according to the specific causes of cancer death or cardiovascular death as relevant data were not available. Fifth, we have compared the baseline characteristics of the included participants with those excluded in the Additional file 1: Table S1. The majority of those excluded were those had cardiovascular disease, diabetes or cancer at baseline. As expected, individuals excluded tended to be older, more often female and less often white, and were more likely to be obese and a smoker. Lastly, dietary fiber intake was assessed at baseline only and fiber intake pattern could have changed during the follow-up period.

In conclusion, in this large nationally representative sample of US adult population, intakes of total fiber, soluble fiber, and insoluble fiber were associated with lower risks of all-cause, cardiovascular and cancer mortality. Given the important role that diet plays in preventing chronic diseases and deaths, nutrition education programs should be implemented in order to promote a healthy diet in the general population.

## Supplementary Information

**Additional file 1: Table S1.** Main characteristics of participants included and not included in this study.

## Data Availability

The data used in this study can be applied from PLCO website (https://cdas.cancer.gov/datasets/plco/).
