# Supplementary material for: Associations between dietary fiber intake and mortality from all causes, cardiovascular disease and cancer: a prospective study
Source: J Transl Med. 2022 Aug 2;20:344. doi: 10.1186/s12967-022-03558-6 (PMC9344643; doi:10.1186/s12967-022-03558-6)
Supplement: Supplementary file 1 — Additional file 1: Table S1. Main characteristics of participants included and not included in this study. [file 12967_2022_3558_MOESM1_ESM.docx]

**Table S1 Main characteristics of participants included and not included in this study**

| Variables | Group | Included participants | Excluded participants | p-value |
| --- | --- | --- | --- | --- |
|  |  | (n=86642) | (n=68255) |  |
| Age (y), mean (SD) |  | 62.1 (5.2) | 63.2 (5.5) | **<0.001** |
| Sex | Female | 39681 (45.8%) | 36997 (54.2%) | **<0.001** |
|  | Male | 46961 (54.2%) | 31248 (45.8%) |  |
| Randomization arm (n, %) | Screen | 44207 (51.0%) | 33236 (48.7%) | **<0.001** |
|  | Control | 42435 (49.0%) | 35009 (51.3%) |  |
| Smoking (n, %) | Never | 42669 (49.3%) | 26599 (42.0%) | **<0.001** |
|  | Current | 8059 (9.3%) | 7994 (12.6%) |  |
|  | Former | 35896 (41.4%) | 28719 (45.4%) |  |
| Education (n, %) | ≤High school | 35863 (41.5%) | 28435 (45.1%) | **<0.001** |
|  | ≥Some college | 50604 (58.5%) | 34657 (54.9%) |  |
| BMI (n, %) | <25.0 kg/m^2^ | 30995 (36.2%) | 19554 (31.5%) | **<0.001** |
|  | ≥25.0 kg/m^2^ | 54510 (63.8%) | 42594 (68.5%) |  |
| Race (n, %) | White, Non-Hispanic | 79281 (91.5%) | 53291 (78.1%) | **<0.001** |
|  | Other | 7361 (8.5%) | 14954 (21.9%) |  |
| Marital status (n, %) | Married | 67838 (78.4%) | 45360 (71.9%) | **<0.001** |
|  | Not married | 18639 (21.6%) | 17749 (28.1%) |  |

y, year; SD, Standard deviation; BMI, body mass index
